# Privacy-Preserving Anonymity for Periodical Releases of Spontaneous Adverse Drug Event Reporting Data: Algorithm Development and Validation

**DOI:** 10.2196/28752

**Published:** 2021-10-28

**Authors:** Jie-Teng Wang, Wen-Yang Lin

**Affiliations:** 1 Department of Computer Science and Information Engineering National University of Kaohsiung Kaohsiung Taiwan

**Keywords:** adverse drug reaction, data anonymization, incremental data publishing, privacy preserving data publishing, spontaneous reporting system, drug, data set, anonymous, privacy, security, algorithm, development, validation, data

## Abstract

**Background:**

Spontaneous reporting systems (SRSs) have been increasingly established to collect adverse drug events for fostering adverse drug reaction (ADR) detection and analysis research. SRS data contain personal information, and so their publication requires data anonymization to prevent the disclosure of individuals’ privacy. We have previously proposed a privacy model called MS(*k*, θ*)-bounding and the associated MS-Anonymization algorithm to fulfill the anonymization of SRS data. In the real world, the SRS data usually are released periodically (eg, FDA Adverse Event Reporting System [FAERS]) to accommodate newly collected adverse drug events. Different anonymized releases of SRS data available to the attacker may thwart our single-release-focus method, that is, MS(*k*, θ*)-bounding.

**Objective:**

We investigate the privacy threat caused by periodical releases of SRS data and propose anonymization methods to prevent the disclosure of personal privacy information while maintaining the utility of published data.

**Methods:**

We identify potential attacks on periodical releases of SRS data, namely, BFL-attacks, mainly caused by follow-up cases. We present a new privacy model called PPMS(*k*, θ*)-bounding, and propose the associated PPMS-Anonymization algorithm and 2 improvements: PPMS+-Anonymization and PPMS++-Anonymization. Empirical evaluations were performed using 32 selected FAERS quarter data sets from 2004Q1 to 2011Q4. The performance of the proposed versions of PPMS-Anonymization was inspected against MS-Anonymization from some aspects, including data distortion, measured by normalized information loss; privacy risk of anonymized data, measured by dangerous identity ratio and dangerous sensitivity ratio; and data utility, measured by the bias of signal counting and strength (proportional reporting ratio).

**Results:**

The best version of PPMS-Anonymization, PPMS++-Anonymization, achieves nearly the same quality as MS-Anonymization in both privacy protection and data utility. Overall, PPMS++-Anonymization ensures zero privacy risk on record and attribute linkage, and exhibits 51%-78% and 59%-82% improvements on information loss over PPMS+-Anonymization and PPMS-Anonymization, respectively, and significantly reduces the bias of ADR signal.

**Conclusions:**

The proposed PPMS(*k*, θ*)-bounding model and PPMS-Anonymization algorithm are effective in anonymizing SRS data sets in the periodical data publishing scenario, preventing the series of releases from disclosing personal sensitive information caused by BFL-attacks while maintaining the data utility for ADR signal detection.

## Introduction

### Motivation

Adverse drug reactions (ADRs) are undesirable side effects of taking drugs. Before hitting the market, a new drug has to undergo a series of clinical trials. Unfortunately, it is hard to find all ADRs in the premarketing stage due to fewer volunteers. Thus, an increasing number of countries have built spontaneous reporting systems (SRSs) to collect adverse drug events (ADEs) to monitor the safety of marketed drugs, such as the FDA Adverse Event Reporting System (FAERS) of the US Food and Drug Administration (FDA) [1], the UK Yellow Card scheme [2], and the MedEffect Canada [3]. Some countries even publish their SRS data sets, for example, US FDA and MedEffect Canada, to the public to facilitate ADR research.

SRS data are a kind of microdata containing personal health information, such as diseases of the patients. Microdata, usually represented in the form of tables of tuples [4], are composed of explicit identifier (*ID*) that can uniquely identify each individual (eg, SSN, name, phone number); quasi-identifier (*QID*) that can be linked with external data to reidentify some of the individuals (eg, sex, age, and ZIP code); sensitive attribute (*SA*) that contains sensitive information, such as disease or salary; and non-SA that falls into none of the above 3 categories. Publishing these data sets would lead to privacy threats. A real case did occur in Canada. A broadcaster successfully reidentified a 26-year-old girl by linking MedEffect Canada and the publicly available obituaries [5]. This case motivated the research by El Emam et al [5], whose findings showed that the MedEffect Canada data exhibit a high risk of identity disclosure.

Generally, simple removal of the identification attributes, such as name, SSN, or phone, has been shown to fail to protect individual privacy [6]. The adversary can still link published data to external data (eg, voter list, through quasi-identification attributes, such as gender, job, age, ZIP code). This calls for the research topic, namely, privacy-preserving data publishing (PPDP), which aims to anonymize raw data before publication. In [7], we pointed out that none of traditional anonymization methods (eg, *k*-anonymity [6], *l*-diversity [8]) is favorable for SRS data sets due to characteristics such as multiple individual records, multivalued SAs, and rare events. Later, we proposed a privacy model called MS(*k*, θ*)-bounding [9] to anonymize SRS data to prevent the disclosure of individual privacy. New events arrive in SRSs continuously in the real world, so countries such as the USA and Canada release SRS data sets periodically, for example, every quarter, to handle this kind of dynamically growing data sets (ie, periodical data publishing). Unfortunately, MS(*k*, θ*)-anonymity is designed for a single static publishing scenario, and is awkward to handle a series of published data sets.

Usually, each ADE record in SRS data contains a CaseID to trace the follow-ups of that event; all records with the same CaseID, located within the same or different periods, refer to the same event. Although someone may regard follow-ups as duplicates of the original case, the situation is somewhat different. Follow-up cases contain complement or correction of the original case. Still, duplicate reports refer to the same case submitted by different reporters, so were misrecorded with different CaseIDs. Follow-ups are easily detected via CaseID, but identifying actual duplicates is challenging, which should be considered a data preprocessing issue. There has been some research studies on detecting actual duplicates in SRS data [10-12]. Most SRS systems such as FAERS, however, provide no deduplication mechanism. We thus ignore this issue. Unfortunately, CaseID provides a useful linkage for the adversary across a series of anonymized data sets to exclude records not belonging to the target, raising the risk of breaching the target’s privacy. For illustration, let us consider 3 consecutive quarters of published SRS data sets in Table 1, each of which satisfies 3-anonymity.

**Table 1:** Three consecutive quarters of published spontaneous reporting system data sets, each satisfying 3-anonymity.

Quarter and CaseID		Sex	Age	Disease
**1**				
	1	Male	[35-40]	Flu
	2	Male	[35-40]	Flu
	3	Male	[35-40]	Fever
	4	Female	[30-35]	HIV
	5	Female	[30-35]	Flu
	6	Female	[30-35]	Diabetes
**2**				
	1	ANY	[30-40]	Flu
	4	ANY	[30-40]	HIV
	7	ANY	[30-40]	Diabetes
	8	Male	[30-35]	Fever
	9	Male	[30-35]	Flu
	10	Male	[30-35]	Diabetes
	11	Male	[30-35]	HIV
	12	Male	[30-35]	Flu
**3**				
	13	Female	[30-35]	Flu
	14	Female	[30-35]	Diabetes
	15	Female	[30-35]	Fever
	16	Female	[30-35]	Flu
	17	Female	[30-35]	Fever
	7	Male	[30-35]	Diabetes
	8	Male	[30-35]	Fever
	18	Male	[30-35]	HIV

### Possible Scenarios

#### Scenario I

Suppose that the adversary learns that his/her neighbor Alice, whose *QID* value is {Female, 32}, suffered from some ADR in Q2. First, the adversary links to Table 1 (quarter 2) through the *QID* of Alice, learning that the record of Alice is in the first *QID* group (CaseIDs 1, 4, and 7). The adversary can then link to the previously published SRS data through the candidate CaseID set {1, 4, 7} and find the record with CaseID=1 and Sex=Male in Table 1 (quarter 1). Because Alice is female, the adversary can exclude CaseID 1 from the candidate CaseID set {1, 4, 7}, changing Table 1 (quarter 2) to 2-anonymous and lifting the confidence of the attacker to identify Alice.

#### Scenario II

Following the previous example, the adversary has known the candidate CaseID set of Alice {4, 7}. The adversary can now use this set to link to subsequently published SRS data and observe a record whose CaseID is 7 in Table 1 (quarter 3). Because the owner of that record is male, the adversary can exclude CaseID 7 from the candidate CaseID set, concluding that the CaseID of Alice in Table 1 (quarter 2) is 4.

#### Scenario III

Suppose that the adversary learns John’s *QID* value is {Male, 33} and the first time that John had an ADR is in Q3. This means that the CaseID of John’s event is a “new CaseID” in Q3 and shall not appear in any previously released data. First, the adversary links to Quarter 3 and learns that the record of John is within the second *QID* group (CaseIDs 7, 8, 18). The adversary can then connect to the 2 previously published SRS data sets through the candidate CaseID set of John {7, 8, 18}, observing 2 matching records whose CaseID are 7 and 8 in Quarter 2. The CaseID of John is neither 7 nor 8, so the adversary concludes that the CaseID of John is 18, ruining the privacy protection embedded by 3-anonymity.

### Background Knowledge and Related Work

#### Privacy Models for Microdata Publishing

Research on PPDP [4] aims to protect released microdata from 2 types of privacy attacks: *record disclosure* and *attribute disclosure*.

Record disclosure, also known as *table linkage attack*, refers to the situation in which the individual identity of a specific tuple that has been deidentified in the published data is reidentified. Although it is hard to prevent table linkage attacks, it is possible to reduce the possibility of identifying victims in a published data. Achievement is the invention of *k*-anonymity [6], which is the most influential privacy model that generalizes the values of *QID* to ensure that each record in published data contains at least *k*–1 other records with the same *QID* value.

Attribute disclosure, also known as *attribute linkage attack*, refers to the situation in which attackers can infer an individual’s sensitive information, even though they fail to perceive the exact record of the victim. Unfortunately, *k*-anonymity is not able to prevent attribute disclosure. Another renowned privacy model called *l*-diversity [8] was thus proposed. The main idea of *l*-diversity is to thwart the adversary’s belief on the probability of the sensitive value by ensuring that each *QID* group contains at least *l* “well-represented” sensitive values, that is, the probability of inferring the sensitive value of the victim will be at most 1/*l*.

#### Privacy Models for Incremental Data Publishing

Most real-world data are not static but dynamically changing, which means that data cannot be published simultaneously but have to be published incrementally [4]. Previously proposed privacy models such as *k*-anonymity and *l*-diversity only focus on single static data publishing, awkward to prevent privacy disclosure in incremental data publishing. Contemporary privacy models for incremental data publishing can be classified into *continuous* or *dynamic* data publishing [4].

#### Continuous Data Publishing

This refers to the scenario in which all data collected so far have to be published even if some of the data have been released before. More precisely, suppose that the data holder had previously collected a set of records *D*_1_ time stamped *t*_1_ and published the anonymized version *R*_1_ of *D*_1_. After collecting a new set of records *D*_2_ time stamped *t*_2_, the data holder will publish *R*_2_ as an anonymized version of all records collected so far, (ie, *D*_1_ ∪ *D*_2_). In general, the published release *R_i_* (*i*≥1) shall be an anonymized version of *D*_1_ ∪ *D*_2_ ∪ ... *D_i_*.

Byun et al [13] first identified the privacy threat under continuous data publishing. They demonstrated possible inference channels by comparing different *l*-diverse releases to explore the sensitive values of victims. They later enhanced their approach by considering both *k*-anonymity and *l*-diverse called (*k*, *c*)-anonymous and exploring more types of adversarial attacks named *cross-version inference*s [14].

Pei et al [15] illustrated that in the continuous data publishing scenario, the adversary can infer some privacy information from multiple releases that have been sanitized by *k*-anonymity. They also proposed an effective method called “monotonic incremental anonymization,” which would progressively and consistently reduce the generalization granularity as the updates arrive to maintain *k*-anonymity.

Fung et al [16] proposed a method to quantify the exact number of records that can be “cracked” by comparing the series of published *k*-anonymous data. The adversary can exclude the cracked records from published data, making the published data no longer satisfy *k*-anonymous. They also presented a privacy model, called *BCF-*anonymity, to measure the anonymous number in published data after excluding the cracked records, and proposed an algorithm to anonymize published data achieving *BCF*-anonymity.

#### Dynamic Data Publishing

This refers to the scenario in which the data holder can insert records into or delete records, or perform both actions, from raw data sets. Suppose that the data holder had collected an initial set of records *D*_1_ in time *t*_1_ and published its anonymized version *R*_1_. During the period [*t*_1_, *t*_2_), the data holder kept collecting new records and inserted them into *D*_1_. Further, the data holder might delete and update some records from *D*_1_, finally obtaining the updated version *D*_2_ of *D*_1_ in *t*_2_. Then, the published release *R*_2_ in *t*_2_ is an anonymized version of *D*_2_. In general, a published release *R_i_* in time *t_i_* shall be an anonymized version of *D_i_*.

Xiao and Tao [17] identified a kind of privacy disclosure called *critical absence*. The adversary can infer victims’ sensitive information by comparing the series of published *l*-diverse data in dynamic data publishing scenarios (only considered insertion and deletion). They proposed a privacy model, called *m*-invariance, to ensure the certain “invariance” of the “signature” of *QID* groups, and an effective method called counterfeited generalization to anonymize published data achieving *m*-invariance.

Bu et al [18] noticed that some sensitive values would be permanent, such as criminal record and some incurable diseases, such as HIV. They showed that *m*-invariance is unable to prevent privacy disclosure when permanent sensitive values are present. Therefore, they proposed an anonymization approach, called *HD-*composition [18], to limit the probability of linkage between individuals and sensitive values not over a given threshold.

On observing *m*-invariance only considers data evolution caused by insertion and deletion, Li and Zhou [19] further presented a counterfeit generalization model named *m*-distinct to support full data evolution (ie, insertion, update, and deletion). Moreover, they observed that attribute updates are seldom arbitrary, with some correlations often existing between the old and the new values. Based on this observation, they assumed that all updates on sensitive values are nonarbitrary. Therefore, *m*-distinct applies the concept of the candidate update set, which is a set of specific sensitive values that can be updated.

Following the work in [19], Anjum et al [20] further assumed that the updates in fully dynamic data publishing are arbitrary, meaning the old values of attributes may not correlate with the new values. They presented a new kind of attack named τ-attack by exploiting the “event list” of an individual. They also proposed a method called τ-safety, an extension of *m*-invariance, to solve the privacy disclosure caused by τ-attack.

He et al [21] presented a new type of attack named *value equivalence attack*, which can exploit the partitioned structure of published data, such as *m*-invariant releases, to obtain sensitive information of individuals. Once the adversary knows the actual sensitive value of an individual, he/she can disclose the sensitive information of the remaining individuals within the same equivalence class. They proposed a graph-based anonymization algorithm, which leverages a min-cut algorithm to prevent the old “value association attack” and the new “equivalence attack.”

Specifically, Bewong et al [22] focused on transactional data. They proposed a new privacy model called *serially preserving*, which requires the posterior probability of any sensitive term to its corresponding population rate bounded by a given threshold. A novel anonymization method (Sanony, which counts on adding counterfeits) was presented to guarantee a new published transactional data set satisfying the required privacy model.

There is another scenario of nonstatic data publishing called *sequential data publishing*. Different vertical projections of the same table on different subsets of attributes are published consecutively in this scenario. Anonymization models and methods for this scenario were first studied in [23] and then further investigated in [24] and [25].

In summary, no contemporary work notices the scenario of periodical data publishing, and no work has been conducted for SRS data anonymization, considering the privacy threat caused by follow-up cases. In this paper, we investigate the privacy threat caused by periodical releases of SRS data and propose anonymization methods to prevent the disclosure of personal privacy information while maintaining the utility of published data.

## Methods

### Publishing Scenario and Privacy Attacks

We first introduce the periodical data publishing scenario and present 3 kinds of privacy attacks for periodically published SRS data sets satisfying MS(*k*, θ*)-bounding. We propose a new privacy model, PPMS(*k*, θ*)-bounding, to protect published SRS data sets from those attacks in the periodical data publishing scenario. We also propose a corresponding anonymization algorithm, namely PPMS-anonymization, that incorporates 2 innovative strategies, *NC*-bounding and *QID*-covering, to prevent the released data sets from privacy attacks caused by follow-up key (ie, CaseID). Two extensions of PPMS-anonymization, PPMS+-anonymization and PPMS++-anonymization, are presented as well, which employ more efficient techniques, including neglecting subsequent coverings and grouping with new cases.

### BFL-Attacks

Typical SRS data, such as FAERS, are usually published periodically and contain follow-up cases, which can be expressed as a new data publishing model named periodical data publishing. Suppose that the data holder previously had collected an initial set of records *D*_1_ in period [*t*_0_, *t*_1_) and published *R*_1_ as an anonymized version of *D*_1_. After collecting a new set of records *D*_2_ during period [*t*_1_, *t*_2_) the attacker wants to anonymize and publish *D*_2_ at time *t*_2_. *D*_2_ may or may not contain some follow-up cases in *D*_1_. Let *R*_2_ denote the anonymized version of *D*_2_. In general, the release *R_i_* published at *t_i_* is an anonymized version of *D_i_* (*i*≥1). Note that for an original case *x*, the life span of its follow-up cases in subsequent releases is not continuous. That is, a follow-up observed in *D_i_* may disappear in *D_i_*_+1_ but show up again in some later release *D_i_*_+_*_j_*, for *j*>1. This makes the periodical publishing scenario distinct from existing scenarios in the literature. First, unlike the situation in dynamic data publishing, *D_i_* is a new set of collections, rather than updated from *D_i_*_–1_. Besides, the existence of follow-up cases is different from the assumption for continuous data publishing (ie, all cases in *D_i_* should be kept in all subsequent releases *D_j_*, for *j>i*)*.* A comparison of the proposed periodical data publishing with dynamic data publishing and sequential data publishing is summarized in Multimedia Appendix 1 (also see Textbox 1).

**Definition 1: QID-cover.**

Consider the *QID* values, *q*_1_ and *q*_2_, of 2 cases. We say *q*_1_ covers *q*_2_, denoted by *q*_1_ 
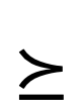
 *q*_2_, if for every attribute *a* in *QID*, *a*(*q*_1_) is equal to or more generalized than *a*(*q*_2_), where *a*(*q*) denotes the value of *q* in attribute *a*.

#### Backward-Attack (B-Attack)

Backward-Attack (*B*-attack) focuses on excluding records from the specific release by exploiting some previous ones (Textbox 2). Scenario I is an example, which occurs when the *QID* value of the old case differs from the background learned by the attacker. As the *QID* values would have been generalized in all published releases, the only way by which *B*-attack can succeed is when the *QID* value of old CaseID fails to cover that of the current CaseID. More precisely, for every target *v*, if in any previous release there exists an old CaseID *i*_old_ corresponding to the candidate CaseID set of *v* such that the *QID* value of *i*_old_ does not cover the *QID* value of *v*, then *i*_old_ would be excluded from the candidate CaseID set of *v*.

**Definition 2: Backward-attack.**

Consider a target *v* to be inferred by the attacker and an anonymized release *R_i_*. Let *q^v^* and *CI* denote the *QID* value and the candidate CaseID set of *v* in *R_i_*, respectively, and *U* be the set of records in all previous releases {*R*_1_, *R*_2_, ..., *R_i_*_–1_} whose CaseID is in *CI*. The *B*-attack will occur if there exists a record *r* in *U* such that the *QID* value of *r*, *q^r^*, does not cover *q^v^*. The set of these excludable records is denoted by *B*.

#### Forward-Attack (F-Attack)

Analogous to *B*-attack, Forward-Attack (*F*-attack) occurs when the *QID* value of the following CaseID differs from the background learned by the attacker (Textbox 3). That is, the *QID* value of a following CaseID in some subsequent releases fails to cover that of the current CaseID. An example is shown in Scenario II. More precisely, for every target *v*, if in any subsequent release there exists a following CaseID *i*_new_ corresponding to the candidate CaseID set of *v* such that the *QID* value of *i*_new_ does not cover the *QID* value of *v*, then *i*_new_ would be excluded from the candidate CaseID set of *v*.

**Definition 3: Forward-attack.**

Consider a target *v* and an anonymized release *R_i_*. Let *q^v^* and *CI* denote the *QID* value and the candidate CaseID set of *v* in *R_i_*, respectively, and *U* be the set of records in all subsequent releases {*R_i_*_+1_, *R_i_*_+2_, ..., *R_c_*} whose CaseID is in *CI*. The *F*-attack will occur if there exists a record *r* in *U* such that the *QID* value of *r*, *q^r^*, does not cover *q^v^*. The set of these excludable records is denoted by *F*.

#### Latest-Attack (L-Attack)

This attack is illustrated in Scenario III. In this example, the attacker knows that the event for the target (John) first appears in Quarter 3. It follows that John’s case (CaseID) is definitely absent in all previously published releases. In general, for every target *v* whose CaseID is first present in some release known by the attacker, *Latest Attack* (*L*-attack) would occur if the candidate CaseID set of *v* contains some old CaseIDs appearing in previous releases (Textbox 4).

**Definition 4: Latest-attack.**

Consider a target *v*. Suppose the attacker learns that the CaseID of *v* first appears in an anonymized release *R_i_*. Let *CI* be the candidate CaseID set of *v* in *R_i_*. The *L*-attack will occur if there exists any case in *CI* whose CaseID appears in some previous releases. The set of these excludable records is denoted by *L*.

### Privacy Model PPMS(k, θ*)-bounding

To prevent *BFL*-attacks, we propose a new privacy model called periodical-publishing multisensitive (*k*, θ*)-bounding, abbreviated as PPMS(*k*, θ*)-bounding (Textboxes 5 and 6).

**Definition 5: Confidence.**

Let *s* be a sensitive value in *SA* and an anonymized release *R_i_*. Given a target *v* with *QID* value *q^v^*, we define the probability that *v* has sensitive value *s* as *conf*(*v* → *s*), which is equal to *σ_s_*(*g*)/|*g*|, where *g* denotes the *QID* group in *R_i_* in which *v* resides and *σ_s_*(*g*) is the number of cases in *g* that contains *s*.

Definition 6: PPMS(k, θ*)-bounding.

Let *S*={*s*_1_, *s*_2_, ..., *s_m_*} be the set of all possible sensitive values in *SA* and θ*=(*θ*_1_, *θ*_2_, ..., *θ_m_*) be the probability thresholds specified by the data holder, where 0≤*θ_j_*≤1, for 1≤*j*≤*m*. We say a series of anonymized releases *R*_1_, *R*_2_, ..., *R_n_* satisfies PPMS(*k*, θ*)-bounding if each *R_i_*, 1 ≤ *i* ≤ *n*, satisfies the following:

1. For every individual *v*, the size of the candidate CaseID set *CI* of *v* in *R_i_* excluding *B*, *F*, and *L* is no less than *k*, that is, |*CI* – (*B*∪*F*∪*L*)| ≥ *k*, and

2. The confidence to infer *v* having any sensitive value *s_j_* ∈ *S* is no larger than *θ_j_*, that is, *conf*(*v* → *s_j_*) ≤ *θ_j_*.

The privacy requirement of Definition 6(1) is to prevent record disclosure while Definition 6(2) is to prevent attribute disclosure. Our model adopts nonuniform thresholds for different sensitive values because different values express different degrees of sensitivity in the real world. For example, the disclosure of a patient with fever is far less sensitive than that of an individual with HIV.

### Anonymization Algorithm

#### Overview

Our algorithm can be summarized as a greedy and clustering approach to divide records into *QID* groups. Viewing each *QID* group as a cluster, we adopted a clustering-based method [26] to build *QID* groups, each of which starts from a randomly chosen record and grows gradually by adding a solo record exhibiting the best characteristic among all candidates. This process repeats until the *QID* group satisfies the “*k*” requirement. Finally, we generalize the *QID* values of all records within the same cluster to the same value.

We adopted 2 metrics, information loss [26] (Textbox 7) and privacy risk (PR) [9] (Textbox 8), to choose the best isolated record. For each evolving *QID* group, the former favors the new record contributing minimal impact to the data utility while the latter quantifies the ratio of sensitive values within the *QID* group to meet the privacy requirement in Definition 6(2).

**Definition 7: Information loss.**

Suppose the *QID* attributes can be separated to 2 different sets, numerical attributes {*N*_1_, *N*_2_, ..., *N_m_*} and categorical attributes {*C*_1_, *C*_2_, ..., *C_n_*}, and each *C_i_* is associated with a taxonomy tree *T_i_*. Let *g* denote a *QID* group (or cluster). The *information loss* (*IL*) [26] of *g* is defined as follows:

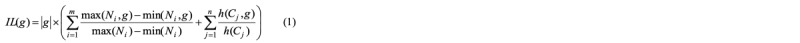

where max(*N_i_*) and min(*N_i_*) denote the maximum and minimum values of attribute *N_i_* in the whole data set, and max(*N_i_*, *g*) and min(*N_i_*, *g*) denote the maximum and minimum values of attribute *N_i_* in *g*. Notation |*g*| is the number of records in *g*, *h*(*C_j_*) the height of the taxonomy tree *T_j_*, and *h*(*C_j_*, *g*) is the height of the generalized value of *C_j_* in *g* in taxonomy tree *T_j_*.

To find a new record *r* to be included in *g*, we choose the one causing the least increase of information loss, which is measured by

Δ*IL*(*g*, *r*)=*IL*(*g* ∪ {*r*}) – *IL*(*g*) **(2)**

Then, the most feasible choice *r_bst_* is

*r_bst_*=argmin*_r_* Δ*IL*(*g*, *r*) **(3)**

In addition, the inclusion of record *r* containing sensitive value *s* that appears in *g* would cause the ratio of *s* in *g* to be over *θ_s_*. As we will derive in Lemma 2, we have to keep the occurrence of *s* in *g*, denoted by *σ_s_*(*g*), under a maximum threshold* η_s_*(*g*) to prevent the confidence of inferring sensitive value *s* in *g* from being larger than *θ_s_*. We thus adopt the *PR_s_* introduced in [9].

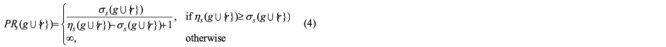

When *η_s_*(*g*∪{*r*}) ≥ *σ_s_*(*g*∪{*r*}), a greater *σ_s_* leads to a larger *PR*_s_. Therefore, Equation 4 favors the new record *r* whose sensitive values are relatively rare in *g*. Because a record may contain more than 1 sensitive value, the PR caused by adding *r* into *g* can be defined as the summation of *PR_s_* over all sensitive values.

**Definition 8: Privacy risk.**

Let *g* denote a *QID* group (or cluster) during the execution of our anonymization algorithm. The PR [9] of adding a new record *r* into *g* is

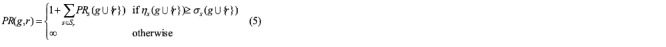

where *s* ∈ *S_r_* and *S_r_* is the set of sensitive values contained in record *r*.

The value of summation of *PR_s_* may be zero, that is, all sensitive values in *r* are new to group *g*. An increment is thus added into PR(*g*, *r*) in Equation 5 to avoid zero PR. The smaller the PR caused by adding *r* into *g*, the more likely *r* will be chosen. If the inclusion of *r* makes the number of records containing *s* in *g* more than the maximally allowed number, PR becomes infinite, so *r* will not be chosen. Finally, we refine Δ*IL* into Δ*IL'* as follows

Δ*IL*ʹ(*g*, *r*)=Δ*IL*(*g*, *r*) × PR(*g*, *r*) **(6)**

and the most feasible choice *r_bst_* is

*r_bst_*=argmin*_r_* Δ*IL'*(*g*, *r*) **(7)**

#### Strategies Against BFL-Attacks

The *NC*-bounding strategy aims to maintain at least “*k*” new CaseID records in each group after excluding all old CaseID records. This is because all old CaseID records may become excludable by exploiting the previous releases, such as *B*-attack and *L*-attack. *QID-*covering is to generalize the *QID* value of records to prevent them from being excluded by *B*-attack and *F*-attack. *NC*-bounding allows the adversary to discover and exclude records not belonging to the target, but enforces the privacy requirement met by the remaining records. *QID-*covering, by contrast, perplexes the adversary to find out excludable records.

#### Strategy for L-Attack

##### Overview

Recall that *L*-attack occurs as the adversary knows the exact published release to which the first ADE of the target *v* belongs. Specifically, let this release be *R_i_*. All old CaseIDs in target *v*’s *CI* set in *R_i_* refer to other targets, which are potentially excluded by the attacker and so should be discounted from forming a valid *QID* group, that is, the size of the *QID* group should be at least *k*. For this reason, we use strategy *NC*-bounding.

##### Example 1

Consider the example in Scenario III. The target *QID* group <Male, [30-35]> in Table 1 (quarter 3) contains 2 old CaseIDs (ie, 7 and 8). We need to add 2 other records with new CaseIDs to make Table 1 (quarter 3) invulnerable to *L*-attack. In this case, all records in the *QID* group <Female, [30-35]> are new cases and the size of <Female, [30-35]> is larger than *k* + 2. We can choose any 2 of them (eg, 16 and 17) into <Male, [30-35]> and generalize the *QID* values accordingly. In general, to defend against *L*-attack, the number of new CaseID records in every *QID* group needs to be no less than *k*.

#### Strategy for B-Attack

##### Overview

Suppose the target *v* is in *R_i_*. *B*-attack means the adversary can link to *R*_1_, *R*_2_, ..., *R_i–_*_1_ through the candidate CaseID set of *v* to exclude those CaseIDs definitely not belonging to target *v*. Note that all of the excludable CaseIDs in *B*-attack are old CaseIDs; thus, the situation is the same as *L*-attack in which all of the old CaseID records have a probability to be excluded. Therefore, the *NC*-bounding strategy used to defend *L*-attack can also be used to secure against *B*-attack. That is, the number of new CaseID records in every *QID* group needs to be larger than or equal to *k* in PPMS(k, θ*)-bounding. In this sense, *L*-attack is similar to *B*-attack, because both of them exploit the previous releases to find excludable CaseIDs. The main difference is that the former needs to know whether the CaseID is old or not, while the latter needs to compare the *QID* values to infer whether the CaseID belongs to the target.

##### Example 2

Consider the example in Scenario I. Similar to the previous example for *L*-attack, we have to include 2 records with new CaseIDs, say 8 and 9, into the *QID* group containing old CaseIDs 1 and 4 in Table 1 (quarter 2), that is, <ANY, [30-40]>, and perform generalization accordingly. Table 2 (quarter 2) shows the resulting anonymized table.

**Table 2:** The anonymized releases against *BFL*-attack for the example in Table 1.

Quarter and CaseID		Sex	Age	Disease
**2**				
	13	Female	[30-35]	Flu
	14	Female	[30-35]	Diabetes
	15	Female	[30-35]	Fever
	16	ANY	[30-40]	Flu
	17	ANY	[30-40]	Fever
	7	ANY	[30-40]	Diabetes
	8	ANY	[30-40]	Fever
	18	ANY	[30-40]	HIV
**3**				
	1	ANY	[30-40]	Flu
	4	ANY	[30-40]	HIV
	7	ANY	[30-40]	Diabetes
	8	ANY	[30-40]	Fever
	9	ANY	[30-40]	Flu
	10	Male	[30-35]	Diabetes
	11	Male	[30-35]	HIV
	12	Male	[30-35]	Flu

#### Strategy for F-Attack

##### Overview

Suppose the target is in *R_i_*. *F*-attack means that the adversary can link to {*R_i_*_+1_, *R_i_*_+2_, ..., *R_n_*} through the candidate CaseID set of target and exclude the CaseIDs that are definitely not referring to the target. Unlike *BL*-attacks, *F*-attack exploits the subsequent releases. The *NC*-bounding strategy works for *BL*-attacks because we can find out which CaseIDs are excludable in the latest raw data set by using previous releases. Unfortunately, because *R_i_*_+1_, *R_i_*_+2_, ..., *R_n_* is not published yet, there is no way to foresee which CaseIDs will be excluded in *R_i_* by employing *F*-attack, causing the *NC*-bounding strategy to be infeasible to defend *F*-attack. By contrast, we know that the adversary can exploit *R_i_* to perform *F*-attack to exclude records in *R*_1_, *R*_2_, ..., *R_i–_*_1_. Therefore, the focus is to protect *R*_1_, *R*_2_, ..., *R_i–_*_1_ from *F*-attack through utilizing *R_i_*. In other words, we have to consider how to anonymize *D_i_* to *R_i_*, making *R_i_* non-exploitable for performing *F*-attack on *R*_1_, *R*_2_, ..., *R_i–_*_1_. By applying the same strategy to all subsequent releases after *R_i_*, that is, *R_i+_*_1_, *R_i+_*_2_, ..., *R_n_*, we protect *R_i_* from *F*-attack.

Let *OC_i_* be the set of old CaseIDs present in at least one of the previous releases *R*_1_, *R*_2_, ..., *R_i–_*_1_. Consider a record *r* whose CaseID is in *OC_i_*. Let *O*={*r*_1_, *r*_2_, ..., *r_p_*} refer to, as in previous releases *R*_1_, *R*_2_, ..., *R_i–_*_1_, the set of records that has the same CaseID as that of *r*. To prevent *F*-attack, we have to ensure that

∀*a* ∈ *QID*, *a*(*r*) 
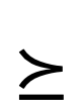

*a*(*r_i_*), for 1 ≥ *i* ≥ *p*.

That is, the *QID* value of *r* should cover that of all *r*’s previous cases.

##### Example 3

Consider the example in Scenario II. To prevent the table published in Quarter 2 from *F*-attack, we have to generalize the 2 records, 7 and 8, in Quarter 3 to cover their corresponding predecessors in Table 1 (quarter 2). This causes the *QID* value of case 7 to become “ANY, [30-40]” and that of case 8 remains unchanged. Because 7, 8, and 18 are in the same *QID* group, we have to generalize their *QID* values into the same value, that is, “ANY, [30-40]”. Finally, if *L*-attack is considered as well, as demonstrated in Example 1, we have to include cases 16 and 17 and finally obtain the result in Table 2 (quarter 2).

##### Lemma 1 (Covering Transitivity)

Consider any 3 records, *r*_1_, *r*_2_, and *r*_3_, with the same CaseID in 3 anonymous releases *R_i_*, *R_j_*, and *R_k_*, *i*<*j*<*k*. If *q^r^*^1^
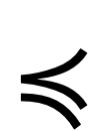
*q^r^*^2^ and *q^r^*^2^
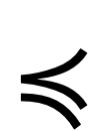
*q^r^*^3^, then *q^r^*^1^
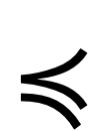
*q^r^*^3^.

Lemma 1 suggests an efficient approach for realizing *QID* covering against *F*-attack. When we are anonymizing *D_i_* to *R_i_*, rather than checking all of the old CaseID records in the previous releases, {*R*_1_, *R*_2_, ..., *R_i–_*_1_}, we only have to search for, starting from *R_i–_*_1_ to *R*_1_, the latest release containing old CaseID records. Once we find that release, we can stop checking the remaining ones.

We next summarize how we can integrate these 2 strategies to meet the privacy requirement in Definition 6(a).

##### Theorem 1

A release *R_i_* anonymized by following strategies of *NC*-bounding and *QID* covering satisfies the requirement of Definition 6(a). For proof, please see Multimedia Appendix 2.

#### Strategy Against Attribute Disclosure

##### Overview

The privacy disclosure caused by *BFL*-attacks not only includes record disclosure but also attribute disclosure. This is illustrated with the following example.

##### Example 4

Consider the 3 consecutive quarters of the 3-anonymous release in Table 1. Recall that in Scenario I the adversary can link to Table 1 (quarter 3) through the *QID* value of Alice {Female, 32} and perceive the *CI* of Alice is {1, 4, 7}, inferring the probability of Alice having any of {Flu, HIV, Diabetes} is 1/3. After employing *B*-attack via Quarter 1, *CI* is reduced to {4, 7}, so the adversary’s confidence that Alice has HIV or diabetes increases to 1/2. He/she can further exclude CaseID 7 from *CI* by performing *F*-attack via Quarter 3 and be 100% sure that Alice has HIV.

Now let us consider how to prevent the attribute disclosure caused by *BFL*-attacks. The basic idea is to control the ratio of sensitive values in each *QID* group to be no greater than the specified threshold. Consider our proposed strategies against *BFL*-attacks stated in the previous section. Let *S_g_*={*s*_1_, *s*_2_, ..., *s_p_*} denote the set of sensitive values in *g* and (*θ*_1_, *θ*_2_, ..., *θ_p_*) the corresponding threshold specified for *S_g_*. We can derive the following occurrence bound for each sensitive value within a *QID* group *g* to meet the required threshold.

##### Lemma 2

For any sensitive value *s*∈*S_g_*, the maximal number of cases in *g* that contains *s* without breaking the associated threshold *θ_s_*, denoted by *η_s_*(*g*), is



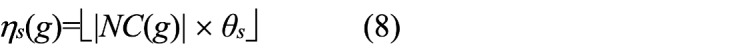



where |*NC*(*g*)| is the number of new CaseIDs in *g*. For proof, please see Multimedia Appendix 3.

#### Algorithm PPMS-Anonymization

Multimedia Appendix 4 presents our algorithm PPMS-Anonymization, which is composed of 3 stages. The first stage aims at finding out old CaseID records and generalizing their *QID* values in advance to achieve *QID-*covering against *F*-attack. Because there may exist multiple individual records [9] in ADE data sets, we follow the *combined record* (or *super record*) concept in [9] to deal with this issue. All records with the same CaseID are combined into a super record before starting to form *QID* groups. Without this process, the records with identical CaseIDs may be divided into different *QID* groups, leading to more substantial deviation in the data quality and perplexing the process of identifying duplicate records while detecting ADR signals.

To find out old CaseID records in *D_i_* and generalize their *QID* values in advance, we check previous releases *R_pre_* from *R_i–_*_1_ to *R_i–x_* (if *i*=1, *R_pre_*=null). Because CaseID is used to trace an event’s follow-ups, there is typically a life span of CaseID, denoted by *x.* The generalization of old CaseID records aims at achieving *QID-*covering against *F*-attack. Because of the transitive property of *QID* value shown in Lemma 1, once we discover an old CaseID record *r'* in any one of the previous releases, we stop checking the remaining earlier releases by using “break” (line 13 in Multimedia Appendix 4) to end the “while loop” (line 8 in Multimedia Appendix 4).

The second stage shown in Multimedia Appendix 5 is activated by calling the procedure *Grouping*, forming as many *QID* groups satisfying PPMS(*k*, θ*)-bounding as possible. Each group begins with a randomly chosen seed record, gradually growing by adding a record with the least Δ*IL'* (defined in Equation 7) until there are at least *k* new CaseID records to achieve the *NC*-bounding strategy. The *OldCaseNum* function returns the number of old CaseID records in a group. A new group then begins with the new record most distinguished from the one just added into the latest group. The above steps are repeated until the remaining records fail to form a group, for example, the number of new CaseID records is less than *k* or the ratio of all sensitive values within the remaining records is higher than the associated threshold (see line 10 in Multimedia Appendix 5).

The last stage is activated by calling the function *Generalization* (Multimedia Appendix 6), which processes the remaining ungrouped records by assigning each of them into the most feasible group that produces the minimal Δ*IL'* to sustain the data utility and satisfy the privacy requirement. Next, the super records will be split back to the original records (the group they belong to remains unchanged). Finally, all records within the same group are generalized into the same *QID* value to satisfy PPMS(*k*, θ*)-bounding.

#### Algorithm PPMS^+^-Anonymization

In this section, we propose an improvement of our PPMS-Anonymization algorithm: PPMS^+^-Anonymization. The idea is to neglect the *QID* covering derived in Lemma 1.

Let *r* be a record in *D_i_* whose CaseID is *c*, *q^r^* the *QID* value of *r*, and *r*_1_, *r*_2_, ..., *r_p_* be the older versions of *r* in the previous releases *R*_1_, *R*_2_, ..., *R_i–_*_1_. To prevent *F*-attack, we have to make *q^r^* cover {*q^r^*^1^, *q^r^*^2^, ..., *q^rp^*}. Although we have exploited the transitivity property in Lemma 1 to avoid checking out all of the old CaseID records in releases *R*_1_, *R*_2_, ..., *R_i–_*_1_, the *QID* value suffers from accumulated generalization. That is, the later the record *r* is published, the more information loss will be caused by generalization. Fortunately, we can limit the accumulated generalization by neglecting all subsequent *QID* coverings.

**Figure 1:**
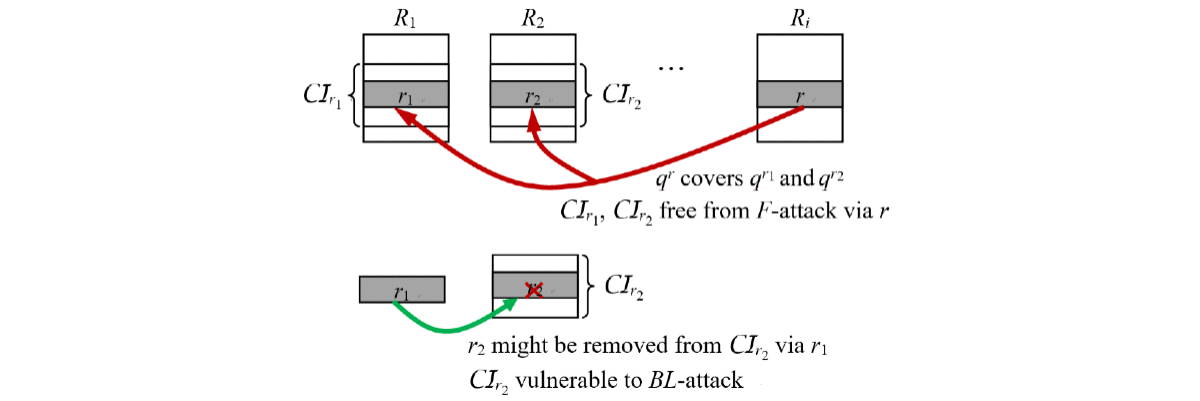
Idea illustration of neglecting subsequent coverings.

The fact is that some of the records protected by *QID-*covering against *F*-attack still can be eliminated by *BL*-attacks. Following the previous discussion, let *r*_1_ be the earliest record with CaseID=*c*. Without loss of generality, assume *r*_1_ resides in *R*_1_. Then clearly, *c* is a new case in *R*_1_, that is, *c* ∈ *NC*(*R*_1_), and will be an old case in all subsequent releases, that is, *c* ∈ *OC*(*R_j_*), 2 ≤ *j* ≤ *i–*1. Remember that all old CaseIDs have the potential to be excluded by *BL*-attacks. So even if we make *q^r^* cover {*q^r^*^2^, *q^r^*^3^, ..., *q^ri^*^-1^} to prevent {*r*_2_, *r*_3_, ..., *r_i_*_-1_} from being excluded by *F*-attack, they can still be excluded by *BL*-attack. This means that generalizing *q^r^* to cover {*q^r^*^2^, *q^r^*^3^, ..., *q^ri^*^-1^} is useless. It suffices to generalize *q^r^* to cover *q^r^*^1^. Figure 1 illustrates this concept.

Multimedia Appendix 7 shows PPMS^+^-Anonymization, the improved version of PPMS-Anonymization in Multimedia Appendix 4 (lines 5-18). For the given record *r*, the modified version seeks *R_i–x_* to *R_i–_*_1_ to find the earliest release in which *r* occurs. Once we find out the earliest old CaseID record *r'*, we stop checking the remaining releases.

#### Algorithm PPMS^++^-Anonymization

##### Overview

In Multimedia Appendix 5, the procedure Grouping works by picking and adding the record with the least Δ*IL'* into the group, overlooking whether the record is a new or an old case in *D'*. We observed that this mixture of new and old cases to form a *QID* group would paralyze the discrimination of Δ*IL* in choosing good candidate records, that is, Equation 7, and cause severe information loss.

Suppose an old CaseID record *r* is picked as the seed to start a new *QID* group *g* in the procedure Grouping. As an old case, the *QID* value of *r* has already been generalized to cover its earliest clone record *r'* in some previous release, meaning that *q^r^* is as coarser as the group in which *r'* resides. Therefore, if there exist some isolated records whose *QID* values are covered by *q^r^*, then adding these records into *g* yields no increase in information loss (ie, Δ*IL*=0)*.* Although this does not affect the information loss of group *g*, it does increase the information loss of the selected record. And in this situation, the Grouping procedure will randomly choose one from those isolated records, disregarding different degrees of information loss brought to these isolated records.

##### Example 5

Consider Table 3. We assume the age attribute has been discretized following the taxonomy tree in Multimedia Appendix 8. The first 3 records form a group starting with the old case record 1, while records 4, 5, and 6 are new cases. Adding any of the 3 isolated records into this group yields no change in the group information loss because all of their *QID* values are covered by record 1. This makes no distinction in choosing the isolated records, but record 6 is the best choice, which exhibits the least data distortion after *QID* generalization.

**Table 3:** An illustration of the problem of *QID* grouping starting with an old case.

*QID* group and isolated records		Sex	Age	Disease
**A forming *QID* group**				
	CaseID 1	ANY	Nonadult	Flu
	CaseID 2	ANY	Nonadult	Flu
	CaseID 3	ANY	Nonadult	Fever
**Isolated records**				
	CaseID 4	Female	Newborn	Fever
	CaseID 5	Male	Preschool	Flu
	CaseID 6	Female	Adolescent	Diabetes

To solve this problem, we avoid mixing new CaseID and old CaseID records in forming *QID* groups. Instead, we separate old CaseID records from *Dʹ* before starting the procedure Grouping, forming possible *QID* groups composed of only new CaseID records. The set of old CaseID records and the remaining new CaseID records are later dealt with by the function Generalization. Multimedia Appendix 9 describes the modification of Multimedia Appendix 4 to realize PPMS^++^-Anonymization, an improvement of PPMS^+^-Anonymization by grouping new cases first.

## Results

### Overview

We designed a series of experiments to examine the effectiveness of our new method in anonymizing a series of periodically released SRS data sets. The proposed PPMS-Anonymization algorithm and its extensions, PPMS^+^-Anonymization and PPMS^++^-Anonymization, were compared with method MS-Anonymization. In this section, we describe the details of each experiment, including the experimental results and our observations.

### Experimental Setup

The data used in our experiment consist of 32 quarterly collections from FAERS, including 2004Q1 to 2011Q4. We used attributes {*Weight*, *Age*, *Gender*} as *QID*, where *Weight* is numerical while the other 2 are categorical, with drug indication (*INDI_PT*) and drug reaction (*PT*) as *SA*. To view *Age* as categorical, we adopted the age taxonomy defined in MeSH [27] (Multimedia Appendix 8). Moreover, we discarded records that have missing values in either *QID* or *SA* attributes.

We respectively performed MS-Anonymization [9] and 3 versions of PPMS-Anonymization, including the original version of PPMS-Anonymization (PPMS), the improved version by incorporating neglecting subsequent coverings (PPMS*^+^*), and the advanced version by employing neglecting subsequent coverings and grouping with new cases (PPMS^++^), to anonymize the selected FAERS data sets, and computed the information loss of 2 series of anonymized data sets. We then imitated the behavior of the adversary, employing *BFL*-attacks to find out all excludable CaseIDs in 2 series of anonymized data sets. After that, we removed all excludable records, and evaluated the risk of record and attribute disclosure of 2 series of anonymized data sets.

We examined 2 aspects of anonymized data sets: information loss and PR. The information loss of an anonymized data set is measured by *normalized information loss* (*NIL*), meaning the average *IL* (using Equation 1) for each attribute of each record.



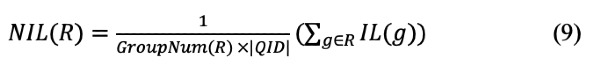



where *R* is an anonymized data set, *g* is a *QID*-group, *GroupNum*(*R*) denotes the number of *QID* groups in *R*, and |*QID*| is the number of attributes in *QID*. This yields *NIL* ranging in [0-1]; the larger the *NIL* is, the more serious is the information loss.

We also used the 2 criteria in [9] to measure the privacy disclosure, *dangerous identity ratio* (*DIR*) and *dangerous sensitivity ratio* (*DSR*); the former measures the ratio of *QID* groups that violate the privacy requirement for protecting record identity, while the latter measures the ratio of *QID* groups that explore sensitive values.

*DIR*(*R*)=*DIGNum*(*R*)/*GroupNum*(*R*) **(10)**

*DSR*(*R*)=*DSGNum*(*R*)/*GroupNum*(*R*) **(11)**

If the number of records in a *QID* group is less than the threshold *k*, we say this group is a *dangerous identity group* (*DIG*). *DIGNum*(*R*) denotes the number of *DIG*s in the anonymized data set *R*. A *QID* group is a *dangerous sensitivity group* (*DSG*) if it contains at least one unsafe sensitive value whose frequency is higher than the associated threshold. *DSGNum*(*R*) denotes the number of *DSGs* in *R*.

To observe the influence of 2 anonymization methods on the strength of ADR signals, we chose from FDA MedWatch [28] all significant ADR rules involving patient demographics such as age or gender conditions and causing withdrawal or warning of the drug. A detailed description of these ADR rules is presented in Table 4. We used the proportional reporting ratio (PRR) [29] description (Multimedia Appendix 10) to measure the strength of ADR signals, which is used by the UK Yellow Card database and UK Medicines and Healthcare products Regulatory Agency (MHRA).

**Table 4:** Selected adverse drug reaction rules from Food and Drug Administration MedWatch.

Drug name and adverse reaction		Demographic condition	Marked year	Withdrawn or warning year
**Avandia**				
	Myocardial infarction Death Cerebrovascular accident	Age>18	1999	2010
**Tysabri**				
	Progressive multifocal leukoencephalopathy	Age>18	2004	2005
**Zelnorm**				
	Cerebrovascular accident	Sex=Female	2002	2007
**Warfarin**				
	Myocardial infarction	Age>60	1940	2014
**Revatio**				
	Death	Age>18	2008	2014

We considered 3 ways of setting θ*. First, we applied a uniform setting on θ*, that is, all confidence thresholds of symptoms were set to the same value (0.2 or 0.4). Then, we used a frequency-based method to determine the threshold of each symptom, which is based on the following idea: The more frequently the symptom occurs, the less sensitive it is. For this purpose, we calculated the average count of symptoms *m* and the corresponding SD. Then we set the confidence thresholds of symptoms whose occurrence is less than *m* – SD, between *m* – SD and *m* + SD, and higher than *m* + SD to 0.2, 0.6, and 1, respectively. Last, we adopted a level-wise confidence setting, which is similar to the frequency setting but conforming to well-recognized medical sensitive terms. All symptoms were classified into 3 levels: high sensitive (*θ*=0.2), low sensitive (*θ*=0.4), and nonsensitive (*θ*=1.0). For this purpose, we followed the setting in [9], choosing the group of symptoms related to AIDS: “Acquired immunodeficiency syndromes” in MedDRA (Medical Dictionary for Regulatory Activities) as high sensitive, 2 groups called “Coughing and associated symptoms” and “Allergies to foods, food additives, drugs and other chemicals” as nonsensitive, and those not belonging to the above groups as low sensitive.

### Results on Anonymization Quality

This section will report the results on information loss and privacy disclosure of MS-Anonymization and our proposed 3 versions of PPMS-Anonymization under 3 different settings of θ*.

#### Uniform Confidence Setting

In this evaluation, we set a uniform threshold (θ*=0.2 and 0.4) to each symptom, that is, the sensitivity of each symptom is the same, and 2 settings of *k* (*k*=5, 10).

##### Information Loss

**Figure 2:**
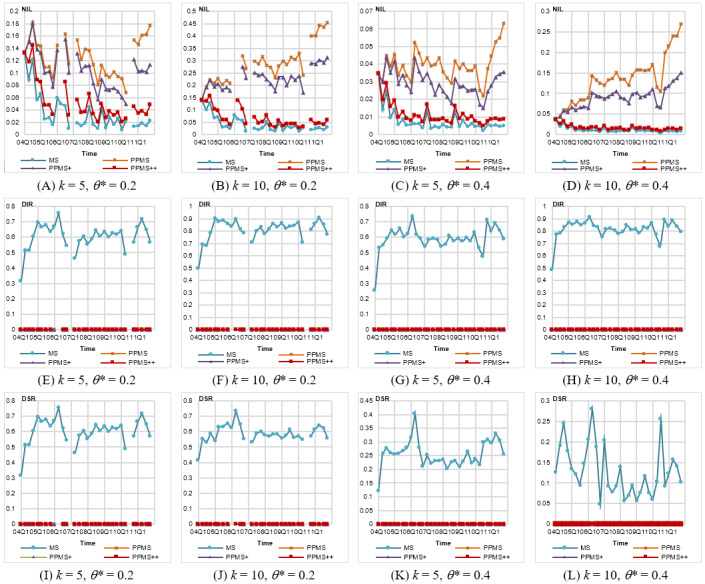
Evaluation on information loss and privacy disclosure for Federal Drug Administration Adverse Event Reporting System (FAERS) data anonymized by different methods with uniform setting of θ*. DIR: dangerous identity ratio, DSG: dangerous sensitivity group, NIL: normalized information loss, PPMS: periodical-publishing multisensitive.

First, we evaluated the information loss. As per the results shown in Figure 2A-D, the general trend is when θ* is lower, the information loss is higher. It is because more records with different sensitive values have to be grouped together to form a valid *QID* group, so more generalization has to be performed. Among the 3 versions of PPMS-Anonymization, PPMS^++^ leads the rank, followed by PPMS^+^ and PPMS, with average improvements of 51% and 59% for PPMS^++^ over PPMS^+^ and PPMS, respectively, as θ*=0.2 and *k*=5, and reaching 78% and 82% for θ*=0.4 and *k*=10. We noticed that as θ*=0.2, some anonymized data sets fail to meet the privacy requirement, that is, 2006Q1, 2006Q2, 2007Q1, and 2010Q3. A further inspection revealed that these data sets contain some highly frequent symptoms. For example, there are 20,467 cases (without missing values) in 2007Q1, and 3877 (18.94%) of them contain “Diabetes Mellitus Non-Insulin-Dependent”. All methods fail in this data set because the minimum bound of that symptom should be 21.00% (3877/18,462, where 18,462 is the number of new cases), so the privacy requirement of 20% cannot be satisfied. In the data set 2010Q3, there are 12,727/56,550 (22.51%) cases containing “Smoking Cessation Therapy,” so no method can meet the privacy requirement. (In 2006Q1 and 2006Q2, the symptom “Myocardial Infarction” is frequent.) In general, the uniform threshold setting is not suitable, especially when some sensitive values are persistent.

##### Record Disclosure

Next, we compared the record disclosure caused by each method. The results are shown in Figure 2E-H. MS-Anonymization exhibits serious record disclosure. The average *DIR*s for *k*=5 and 10 are 0.61 and 0.8, respectively, meaning over half of *QID* groups are *DIG*s. Besides, the *DIR* of MS-Anonymization increases as *k* is larger. This is because a larger *k* leads to less number of groups and so a higher ratio of groups containing old cases, increasing the risk of *QID* groups becoming dangerous. It is noteworthy that the *DIR*s of 3 versions of PPMS-Anonymization are all 0. The reason is that our method guarantees free of record disclosure and the *DIR* metric is not dependent on different settings of θ*.

##### Attribute Disclosure

Finally, we present the results on the *DSR* metric. The results are shown in Figure 2I and J. MS-Anonymization yields very high *DSRs*, 0.6 on average, for lower θ* values (*θ*=0.2). This is because a lower *θ* is more likely to cause the number of symptoms close to its maximal allowed number in the *QID* groups, especially for high-frequent symptoms. Thus, the action of excluding records is more likely to cause the violation of θ* and so leads to relatively higher *DSR*s, such as 2006Q1, 2006Q2, 2007Q1, and 2010Q3. For example, the maximal symptom frequencies in 2006Q4 and 2010Q1 are only 8.1% and 9.1%, respectively, relatively smaller than θ*=0.2 or 0.4, so the *DSRs* of these 2 releases are relatively lower than other releases. This again demonstrates that the uniform threshold setting is not feasible. The setting of *k* also influences the *DSRs* yielded by MS-Anonymization. A larger *k* not only causes higher maximal allowed numbers of symptoms in *QID* groups but also reduces the change in the ratio of symptoms when some records are excluded. Compared with MS-Anonymization, all 3 versions of PPMS-Anonymization yield zero *DSR* value in all data sets, except 2006Q1, 2006Q2, and 2007Q, showing our method can protect data from attribute disclosure caused by *BFL*-attacks.

#### Frequency-Based Confidence Setting

Two different settings of *k* (5 or 10) are considered. The results on *DIR* are omitted because they are similar to those generated by uniform setting, that is, MS-Anonymization generates large *DIR*s while our PPMS-Anonymization yields zero *DIR*.

##### Information Loss

**Figure 3:**
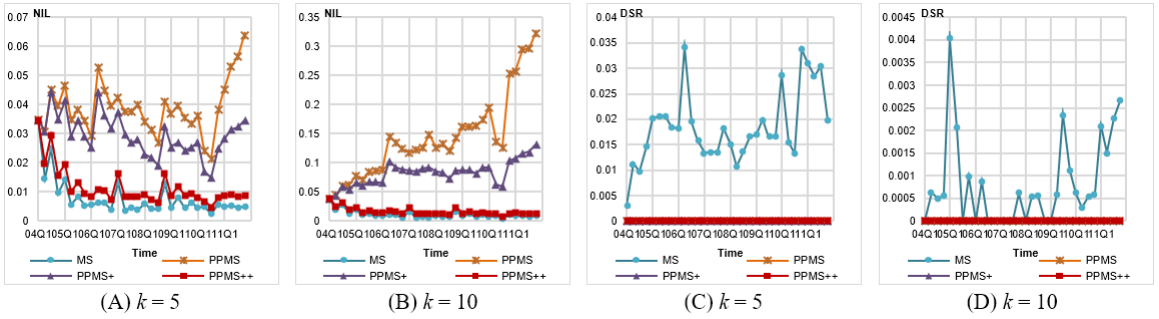
Evaluation on information loss and privacy disclosure for Federal Drug Administration Adverse Event Reporting System (FAERS) data anonymized by different methods with frequency-based setting of θ*. DSR: dangerous sensitivity ratio, NIL: normalized information loss, PPMS: periodical-publishing multisensitive.

As shown in Figure 3A and B, the *NIL*s generated by each method are better than those under the uniform setting. It is not surprising because this more flexible setting easily allows the methods to choose the closer new record to be added during *QID* group construction. Similar to those observed for the uniform setting, PPMS^++^ significantly outperforms PPMS^+^ and PPMS, yielding *NILs* less than 0.05 for *k* =5 and 0.15 for *k* =10.

##### Attribute Disclosure

As shown in Figure 3C and D, all data sets anonymized by PPMS-Anonymization are free of attribute disclosure (ie, zero *DSR*). The *DSR*s of MS-Anonymization are very small compared with those in previous settings. It is because those *DSG*s in the previous experiments are caused by high frequent symptoms, whose thresholds, however, are set to 1 in this experiment. In FAERS data, there are more than 20,000 different symptoms. It is hard to determine a suitable threshold for each of them without background knowledge. Therefore, the frequency-based method is a convenient and reasonable way to deal with this issue. This also demonstrates the value of allowing nonuniform settings in our model.

#### Level-Wise Confidence Setting

Again, 2 different *k* (5 and 10) settings are considered, and for the same reason, we omit the results on *DIR*.

##### Information Loss

**Figure 4:**
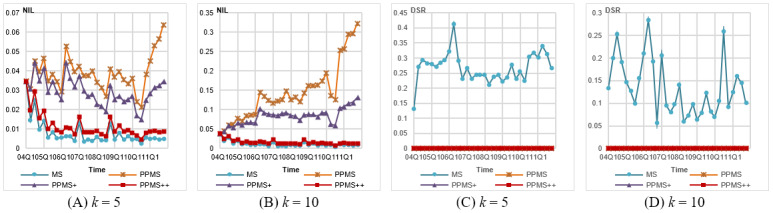
Evaluation on information loss and privacy disclosure for Federal Drug Administration Adverse Event Reporting System (FAERS) data anonymized by different methods with level-wise setting of θ*. DSR: dangerous sensitivity ratio, NIL: normalized information loss, PPMS: periodical-publishing multisensitive.

Figure 4A and B shows that although PPMS and PPMS^+^ yield more information loss than that by MS-Anonymization, PPMS^++^ behaves comparably to MS-Anonymization. The *NIL*s are very similar to those under the frequency-based setting.

##### Attribute Disclosure

The results in Figure 4C and D show that all 3 versions of PPMS-Anonymization cause no attribute disclosure (with zero *DSRs*), but large *DSRs* are observed for MS-Anonymization. We can see that the *DSRs* of MS-Anonymization in some quarters are relatively higher, just similar to the results in Figure 2K and L and Figure 3C and D.

### Influence on ADR Signals

#### Selected Signals

In this experiment, we inspected variation on the strength of observed ADR signals shown in Table 4 between before and after anonymization. Because some signals exhibit similar performance, we only show 3 representatives with different demographic conditions, that is, the signals related to Avandia, Zelnorm, and Warfarin, which are shown as follows:

R1: Avandia, Age>18 → Myocardial infarction

R2: Zelnorm, Sex=Female → Cerebrovascular accident

R3: Warfarin, Age>60 → Myocardial infarction

We calculated its occurrences, PRRs, and compared the values with the original values for each signal. We omit the results for uniform setting θ*=0.4 and level-wise setting because similar results were observed for uniform setting θ*=0.2 and frequency-based setting, respectively.

To highlight the impact of anonymization on rare events, we set PRR=0 when *a*<3, where *a* denotes the number of reports that satisfy the specific ADR rule. The threshold *a*≥3 follows Evans et al [29], who investigated a group of newly marketed drugs and suggested that the minimum criteria for a signal are *a*≥3 and PRR>2.

**Figure 5:**
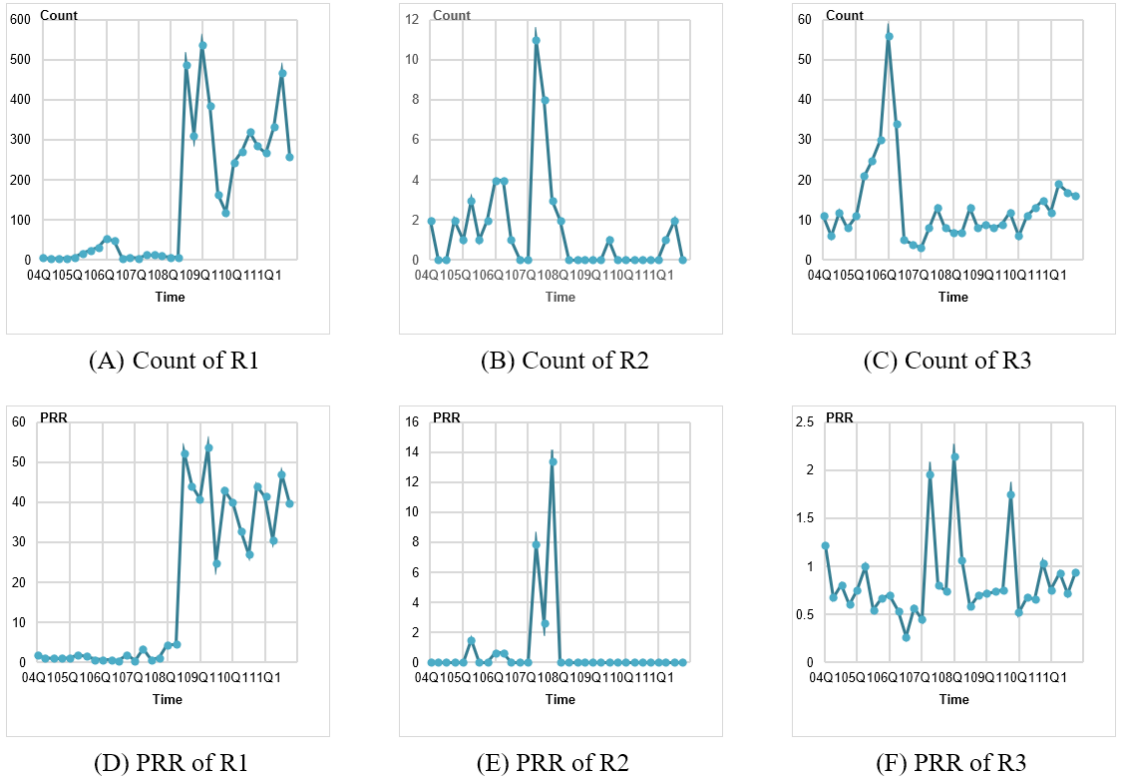
The original counts and proportional reporting ratios (PRRs) of rules R1, R2, and R3.

The original count and PRR of these 3 rules are shown in Figure 5. Rule R1 is a signal with an extremely high occurrence and significant strength, rule R2 is the one with the relatively small occurrence and medium strength, while R3 represents medium occurrence and relatively little strength.

#### Signal Occurrence Variation

**Figure 6:**
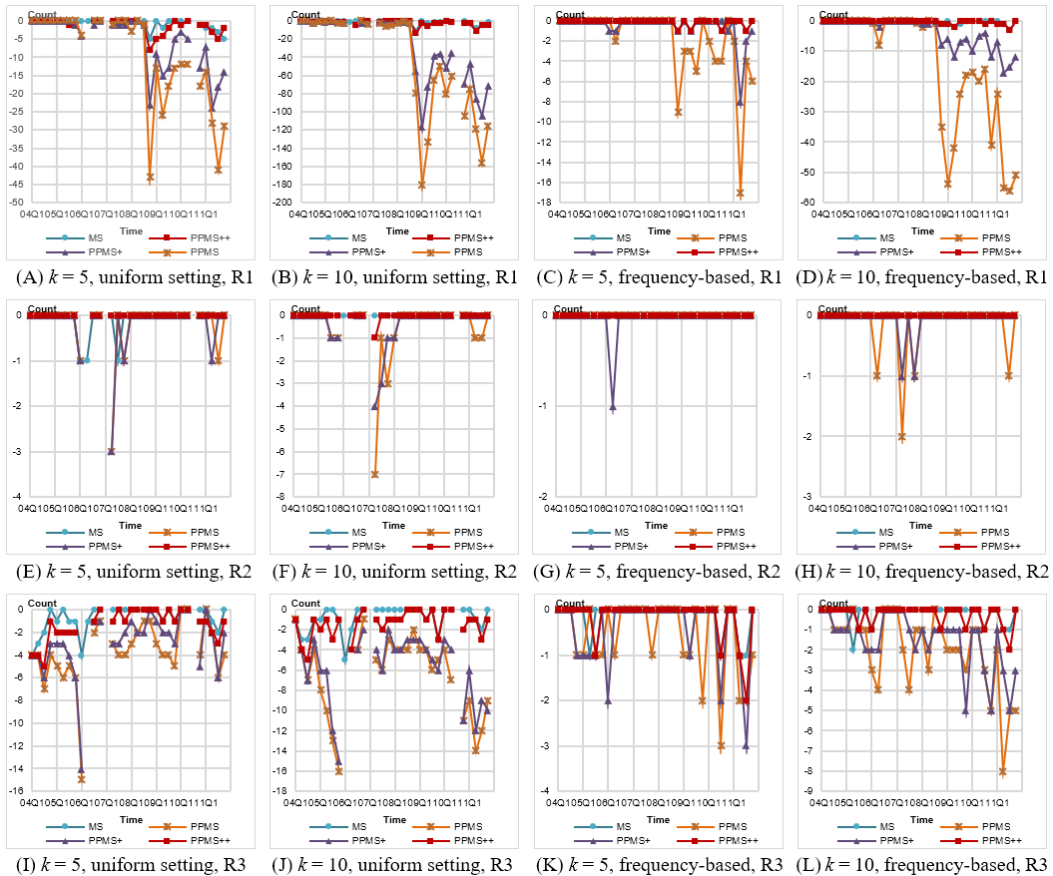
Variations in signal count for different anonymization methods under uniform and frequency-based settings of θ*. PPMS: periodical-publishing multisensitive.

We first evaluated the variation of signal occurrence (count) caused by anonymization. The results are shown in Figure 6. Notice that there is no result for several quarters (eg, 2007Q1, 2010Q3) under the uniform setting. The reason is the same as that for information loss. Generally, the variation yielded by frequency-based setting is much less than that by uniform setting, and a larger *k* causes more missing counts. For signals with extremely high occurrence like R1, the variation can be substantial; for example, it reaches 180 for PPMS with *k*=10 and uniform confidence setting. In the same case, our PPMS^++^ exhibits outstanding performance, only causing variation of less than 10. We also note that some quarters are suffering significant count variation for rule R2 (Figure 6E-H). This is because the taxonomy of Gender is relatively flat, composed of only 2 levels. Once the gender of a report satisfying this rule is generalized, it will become “Any” and increase the missing count of this rule. For example, in Figure 6F, when *k*=10, 7 of 11 counts are missing in 2007Q2 for PPMS. In fact, when *k*=10, the ratio of reports with Gender=Any is at least 25% and 45% from 2010Q4 to 2011Q4 for PPMS^+^ and PPMS, respectively, which causes serious bias on the count of ADR rule. By contrast, as shown in Figure 6G and H, the frequency-based setting exhibits lower missing count. The overall situation shows that PPMS^++^ significantly outperforms PPMS and PPMS^+^, and demonstrates comparable results with MS-Anonymization.

#### Signal Strength Variation

**Figure 7:**
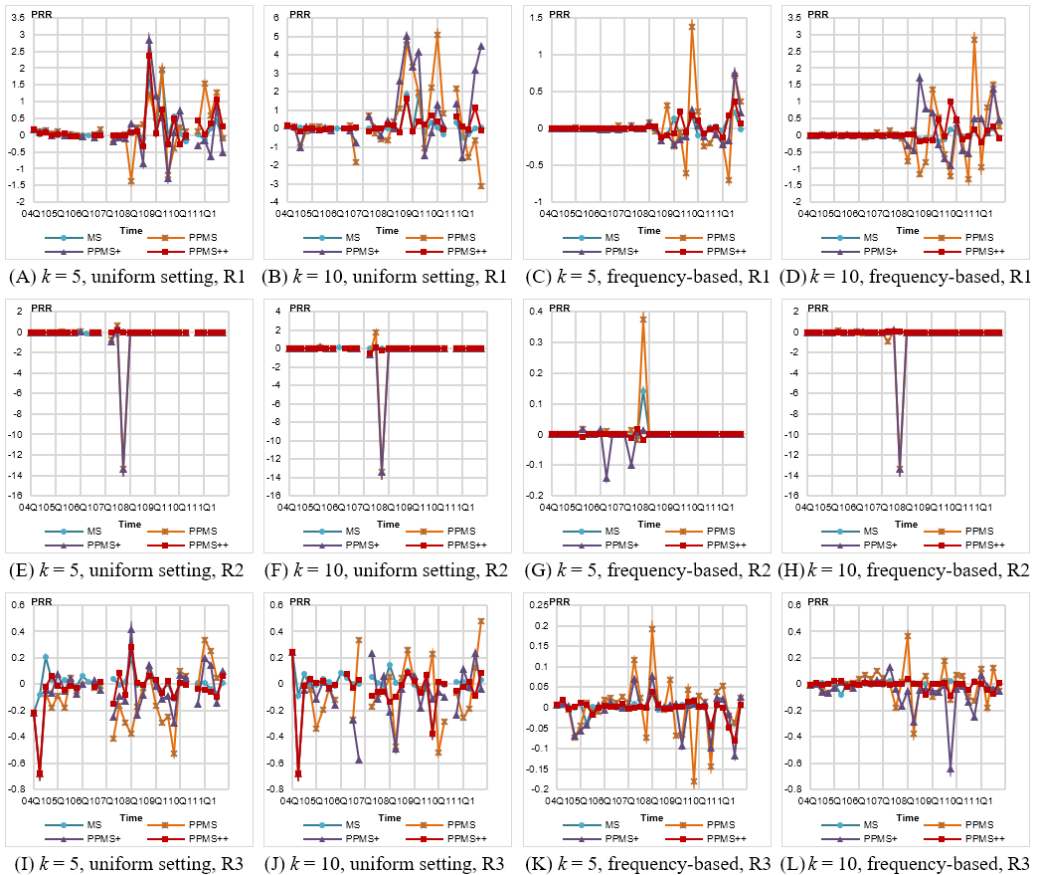
Variations in signal strength (proportional reporting ratio [PRR]) for different anonymization methods under uniform and frequency-based settings of θ*. PPMS: periodical-publishing multisensitive.

Figure 7 shows the results on the PRR difference. Similar to that observed for occurrence variation, the frequency-based setting yields more negligible PRR difference than that by uniform setting. For rule R1 with enormous strength, the PRR variation is significantly higher than those for rules R2 and R3. The variations caused by PPMS and PPMS^+^ fluctuate seriously, sometimes much higher, reaching 5 for *k*=10 and uniform setting of θ*; PPMS^++^ exhibits relatively small variation under the same situation. For rule R2 with attributes of flat taxonomy, we observe a similar phenomenon. Specifically, a sharply significant variation, reaching –14 (Figure 7E, F, and H), is observed in 2007Q4 for PPMS and PPMS^+^. This is because the *a* value for computing PRR is less than 3. We observe that the original count of this rule in 2007Q4 (Figure 5B) is 3 and its original PRR (Figure 5E) is 13.39. This means that this rule is a rare event with high strength. Any missing count of this rule causes value *a* to be less than 3 and the PRR will become 0, invalidating this rule. This situation demonstrates the impact of generalization on rare but significant ADR rule, especially for attributes with shallow generalization levels such as Gender, which will hinder or delay the discovery of ADR signals.

## Discussion

### Principal Results

In this paper, we have introduced the periodical publishing scenario usually adopted for publishing SRS data. We have presented 3 kinds of attacks, *BFL*-attacks, which exploit the CaseID of records to link the same cases in the series of releases to crack the anonymization by excluding the nontargets to improve the confidence to hit the record target or the sensitive value.

To prevent the record and attribute disclosure caused by *BFL*-attacks, we have presented a new model called PPMS(*k*, θ*)-bounding. We have also proposed an algorithm called PPMS-Anonymization to anonymize the raw SRS data set achieving the privacy requirement of PPMS(*k*, θ*)-bounding. Two enhancements of PPMS-Anonymization, PPMS^+^-Anonymization and PPMS^++^-Anonymization, have also been presented.

To evaluate the performance of our method, we conducted several experiments with different settings on privacy threshold, from 3 various aspects of evaluation, including information loss, PR, and bias on signal strength. The results showed that our proposed anonymization method, especially PPMS^++^-Anonymization, can effectively prevent *BFL*-attacks caused by follow-up cases across a series of SRS data sets, guarantee the privacy requirement with controlled loss of data utility, and maintain the usability of anonymized SRS data set for ADR detection, especially for frequency-based threshold setting and level-wise setting.

### Limitations

Fostering the development of new detection methods and early discovery of suspected ADR signals is the main driving force for many organizations such as the US FDA to release their SRS data sets to the public. By contrast, evaluating each individual case safety report (ICSR) is necessary for investigating hypothetical signals generated from the SRS data. Unfortunately, due to national privacy regulations such as the Health Insurance Portability and Accountability Act (HIPPA) Privacy Rule [30], some specified individual identifiers and narrative were removed from the published FAERS data (following the safe harbor method in Section 164.514 [30]). A recent work [31] showed that the absence of personal details would significantly affect the assessment of each ICSR. In this context, the published SRS data alone cannot fulfill the purpose of ICSR evaluation. We endeavor to develop an effective privacy protection method for the partially deidentified SRS data (eg, FAERS) without sacrificing the data utility for aggregative disproportionality analysis of suspected ADR signals. How to protect the sharing and access of raw SRS data containing all individually identifiable health information is beyond the scope of this study. Instead, the SRS data organization should provide advanced security schemes, including technical or nontechnical [32], to ensure the confidentiality, integrity, and availability of the protected health information for authorized users, as enforced by the HIPPA Security Rule [33], which requires a good threat analysis modeling [34] before the system design.

### Comparison With Prior Work

This paper is an extended version of our paper presented at IEEE *ICDE’17* [35]. Some new material has been added to clarify the design of the proposed PPMS-Anonymization and its improvement (PPMS+-Anonymization), including the design of the function Generalization (Multimedia Appendix 6), Multimedia Appendix 7, and Figure 1. A significantly more efficient version, PPMS++-Anonymization, is proposed. A new way of confidence threshold setting, level-wise setting, was evaluated. Additional more ADR signals were inspected. All experiments were reconducted to include the new version (PPMS++-Anonymization). Overall, PPMS++-Anonymization ensures zero PR on record and attribute linkage, while exhibits 51%-78% and 59%-82% improvements on information loss over PPMS+-Anonymization and PPMS-Anonymization, respectively, and significantly reduces the bias of ADR signal. For example, under the frequency setting, the maximum count bias and PRR bias were reduced from 56 to 3 and 13.4 to 0.1, respectively.

Based on our work [35], Huang et al [36] proposed 2 new attacks, *MD*-attack (Medicine Discontinuation attack) and *SS*-attack (Substantial Symptom attack). *MD*-attack assumes the attacker knew when the target stopped his/her treatment, that is, the quarter in which the target’s follow-up record discontinues, while *SS*-attack regards a *QID* group with a substantial amount of adverse reactions risky. Both types of attacks, however, suffer some actuality problems. First, the authors overlooked the phenomenon that an individual’s follow-up records may discontinue for some quarters and reappear in the next quarter. This life span discontinuity of follow-up cases is unpredictable and will thwart the justness of *MD*-attack and the anonymization algorithm. The problem for *SS*-attack is whether knowing someone having many adverse reactions does cause a privacy breach, which needs more convincing evidence. Besides, *SS*-attack is not related to periodical releases of SRS data.

### Conclusions

In summary, our PPMS(*k*, θ*)-bounding and PPMS-Anonymization can anonymize SRS data sets in the periodical data publishing scenario, preventing the series of releases from the disclosure of sensitive personal information caused by *BFL*-attacks.

The *BFL*-attacks caused by the existence of CaseID in SRS data is not a particular case in health data. Other types of medical data contain similar features, for example, electronic health records, a digital version of a patient’s paper chart composed of more private information than SRS data. As far as we know, it contains an attribute called patient ID which is similar to CaseID and so may be vulnerable to *BFL*-attacks. We will study this shortly. Some more challenging extensions of this topic include the study of incremental anonymization of data sets published in a cloud environment [37,38] and handling a large amount of missing values in SRS data [39]. Recently, the emerging differential privacy [40-42] has been widely recognized as a more rigorous privacy protection method [43]. Our recent work [44] on integrating differential privacy to anonymize a single release of SRS data has shown promising results. We are currently synergizing the differential privacy to our PPMS(*k*, θ*)-bounding to yield a better protection scheme.

## Appendix

Multimedia Appendix 1 A summary of privacy models for incremental data publishing.

Multimedia Appendix 2 Proof of Theorem 1.

Multimedia Appendix 3 Proof of Lemma 2.

Multimedia Appendix 4 PPMS-Anonymization.

Multimedia Appendix 5 Procedure Grouping.

Multimedia Appendix 6 Function Generalization.

Multimedia Appendix 7 Modification of PPMS-Anonymization to realize PPMS+-Anonymization.

Multimedia Appendix 8 The taxonomy tree of Age.

Multimedia Appendix 9 Modification of PPMS-Anonymization to realize PPMS++-Anonymization.

Multimedia Appendix 10 Description of proportional reporting ratio.

## Abbreviations

ADE: adverse drug event
ADR: adverse drug reaction
DIG: dangerous identity group
DIR: dangerous identity ratio
DSG: dangerous sensitivity group
DSR: dangerous sensitivity ratio
FAERS: FDA Adverse Event Reporting System
FDA: Food and Drug Administration
HIPPA: Health Insurance Portability and Accountability Act
MedDRA: Medical Dictionary for Regulatory Activities
MHRA: UK Medicines and Healthcare products Regulatory Agency
NIL: normalized information loss
PPDP: privacy-preserving data publishing
PPMS: periodical-publishing multisensitive
PRR: proportional reporting ratio
QID: quasi-identifier
SA: sensitive attribute
SRS: spontaneous reporting system
