# Supplementary material for: Privacy-Preserving Anonymity for Periodical Releases of Spontaneous Adverse Drug Event Reporting Data: Algorithm Development and Validation
Source: JMIR Med Inform. 2021 Oct 28;9(10):e28752. doi: 10.2196/28752 (PMC8587328; doi:10.2196/28752)
Supplement: Multimedia Appendix 1 [file medinform_v9i10e28752_app1.pdf]

Publishing Scenario	Subsequent releases D_i and D_{i+1}	Subsequent anonymized releases R_i and R_{i+1}	Privacy Model	Attack Issue	Type of Privacy Threat	
					Record Disclosure	Attribute Disclosure
**Continuous**						
	$D_i \subset D_{i+1}$	$R_i = A(D_i)$ $R_{i+1} = A(D_i \cup D_{i+1})$	(k, c)-anonymity	Cross inferences	✓	✓
			Incremental k-anonymity	Record identity	✓	
			BCF-anonymity	*BCF*-attacks	✓	
**Dynamic**						
	D_{i+1} is an updated version of D_i by record insertion and/or deletion	$R_i = A(D_i)$ $R_{i+1} = A(D_{i+1})$	m-invariance	Critical absence		✓
			HD-composition	Permanent sensitive values		✓
			m-distinct	Correlation of non-arbitrary update		✓
			m-invariance	Equivalence attacks		✓
			τ-safety	τ-attacks		✓
**Periodical**						
	D_{i+1} is not updated from D_i, but D_{i+1} may contain some follow-up cases in D_i	$R_i = A(D_i)$ $R_{i+1} = A(D_{i+1})$	PPMS(k, θ^*)-bounding	*BFL*-attacks	✓	✓
