# Supplementary material for: Privacy-Preserving Anonymity for Periodical Releases of Spontaneous Adverse Drug Event Reporting Data: Algorithm Development and Validation
Source: JMIR Med Inform. 2021 Oct 28;9(10):e28752. doi: 10.2196/28752 (PMC8587328; doi:10.2196/28752)
Supplement: Multimedia Appendix 2 [file medinform_v9i10e28752_app2.pdf]

Proof. Suppose the adversary link the *QID* value of the target v to R_i ($1 \leq i \leq n$) and find that v is in a *QID*-group g. Let CI be the set of all CaseIDs of records in g, OC the set of all old CaseIDs, and NC the set of new CaseIDs, $OC \cup NC = CI$. Assume B, F, and L represent the set of excludable CaseIDs in CI induced by B-attack, F-attack, and L-attack, respectively. According to our strategy for *BL*-attacks, both B and L equal to OC and $|NC| \geq k$, while for F-attack our *QID*-covering strategy has made all records non-excludable so F is empty. After excluding all excludable CaseIDs from CI, we have $|CI - (B \cup F \cup L)| = |CI - (OC \cup OC \cup \varnothing)| = |NC| \geq k$, which satisfies the privacy requirement of Definition 6(1).
