# Supplementary material for: Privacy-Preserving Anonymity for Periodical Releases of Spontaneous Adverse Drug Event Reporting Data: Algorithm Development and Validation
Source: JMIR Med Inform. 2021 Oct 28;9(10):e28752. doi: 10.2196/28752 (PMC8587328; doi:10.2196/28752)
Supplement: Multimedia Appendix 3 [file medinform_v9i10e28752_app3.pdf]

Proof. Let $\eta_s(B \cup F \cup L)$ denote the number of excludable cases in g containing sensitive value s. After *BFL*-attacks the confidence to infer v having s is

$$\frac{\eta_s(g) - \eta_s(B \cup F \cup L)}{|CI - (B \cup F \cup L)|}$$

We have derived the maxima of $B \cup F \cup L$ is $OC(g)$, yielding the smallest denominator, $|NC(g)|$, of the above equation. So, the maximum value of the above equation occurs when $\eta_s(B \cup F \cup L) = 0$, i.e., all excludable cases do not contain sensitive value s. To prevent the disclosure confidence of s over θ_s, we need

$$\frac{\eta_s(g)}{|NC(g)|} \leq \theta_s$$

The lemma then follows.
