# Supplementary material for: Privacy-Preserving Anonymity for Periodical Releases of Spontaneous Adverse Drug Event Reporting Data: Algorithm Development and Validation
Source: JMIR Med Inform. 2021 Oct 28;9(10):e28752. doi: 10.2196/28752 (PMC8587328; doi:10.2196/28752)
Supplement: Multimedia Appendix 4 [file medinform_v9i10e28752_app4.pdf]

Input: The original dataset D_i, the maximal checking span x, the previous releases $R_{pre} = \{R_{i-x}, \ldots, R_{i-1}\}$, confidence threshold θ^*, and parameter k

Output: An anonymized dataset R_i satisfying PPMS(k, θ^*)-bounding

```
G ← {};    // The set of all anonymized QID-groups
OC ← {}; // The set of all old-CaseIDs
combine records with the same CaseID into one super record per CaseID;
D' ← the set all super records in D_i;
if R_pre != null then
    for each record r in D' do    // Stage 1
        counter ← i – 1;
        while counter ≥ i – x do
            if CaseID cid of r appears in R_counter then
                OC ← OC ∪ {cid};
                r' ← the record in R_counter whose CaseID = cid;
                generalize the QID value of r to cover that of r';    // QID-covering
                break;
            end if
            counter ← counter – 1;
        end while
    end for
end if
Grouping(D', G, OC, k, θ*);    // Stage 2
R_i ← Generalization(D', G, k, θ*);    // Stage 3
return R_i;
```
