# Supplementary material for: Privacy-Preserving Anonymity for Periodical Releases of Spontaneous Adverse Drug Event Reporting Data: Algorithm Development and Validation
Source: JMIR Med Inform. 2021 Oct 28;9(10):e28752. doi: 10.2196/28752 (PMC8587328; doi:10.2196/28752)
Supplement: Multimedia Appendix 5 [file medinform_v9i10e28752_app5.pdf]

Input: D', G, OC, k, θ^*

Output: D', G

```
r ← a randomly chosen record in D';
counter ← 1;
repeat
    create an empty group g_counter into G;
    g_counter ← g_counter ∪ {r};
    remove r from D';
    g_size ← 1;
    while g_size – OldCaseNum(g_counter, OC) < k do   // NC-bounding strategy
        r_bst ← argmin_{r∈D'} ΔIL'(g_counter, r); // using Eq. (7)
        if r_bst = null then   //such as |D'| = 0 or all ΔIL'(g_counter, r) = ∞
            add all records in g_counter into D';
            remove g_counter from G;
            return;
        end if
        g_counter ← g_counter ∪ {r_bst};
        remove r_bst from D';
        g_size ← g_size + 1;
    end while
    r ← the farthest record from r_bst in D';
    counter ← counter + 1;
until ∞
```
