# Supplementary material for: Privacy-Preserving Anonymity for Periodical Releases of Spontaneous Adverse Drug Event Reporting Data: Algorithm Development and Validation
Source: JMIR Med Inform. 2021 Oct 28;9(10):e28752. doi: 10.2196/28752 (PMC8587328; doi:10.2196/28752)
Supplement: Multimedia Appendix 6 [file medinform_v9i10e28752_app6.pdf]

Input: *D'*, *G*, *k*, θ^*

Output: An anonymized dataset *R*

```
for each record r in D' do
    g_best ← argmin_{g∈G} ΔIL'(g, r);
    add r into g_best;
    remove r from D';
end for
split super records back to original records;
for each g ∈ G do
    generalize all records in g into the same QID value;
end for
R ← all records in G;
return R;
```
