# Supplementary material for: Privacy-Preserving Anonymity for Periodical Releases of Spontaneous Adverse Drug Event Reporting Data: Algorithm Development and Validation
Source: JMIR Med Inform. 2021 Oct 28;9(10):e28752. doi: 10.2196/28752 (PMC8587328; doi:10.2196/28752)
Supplement: Multimedia Appendix 7 [file medinform_v9i10e28752_app7.pdf]

```
if R_pre != null then
    for each record r in D' do
        counter ← i – x;
        while counter ≤ i – 1 do
            if CaseID cid of r appears in R_counter then
                OC ← OC ∪ {cid};
                r' ← the record in R_counter whose CaseID = cid;
                generalize the QID value of r to cover that of r';
                break;
            end if
            counter ← counter + 1;
        end while
    end for
end if
```
