# Supplementary figures and images for: Privacy-Preserving Anonymity for Periodical Releases of Spontaneous Adverse Drug Event Reporting Data: Algorithm Development and Validation
Source: JMIR Med Inform. 2021 Oct 28;9(10):e28752. doi: 10.2196/28752 (PMC8587328; doi:10.2196/28752)

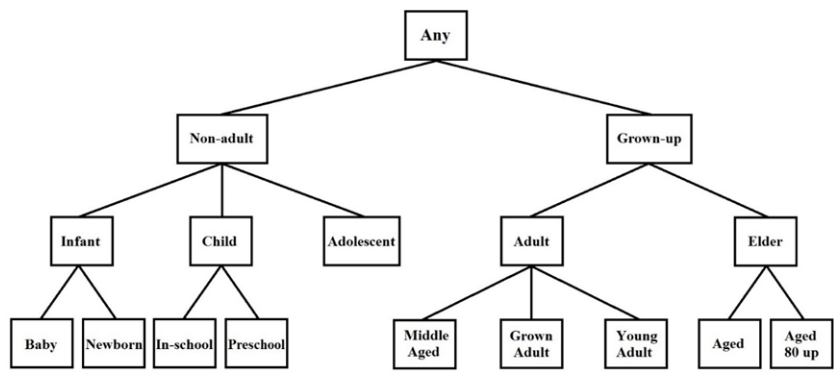

Supplement: Multimedia Appendix 8 [file medinform_v9i10e28752_app8.pdf]
