# Supplementary material for: Privacy-Preserving Anonymity for Periodical Releases of Spontaneous Adverse Drug Event Reporting Data: Algorithm Development and Validation
Source: JMIR Med Inform. 2021 Oct 28;9(10):e28752. doi: 10.2196/28752 (PMC8587328; doi:10.2196/28752)
Supplement: Multimedia Appendix 9 [file medinform_v9i10e28752_app9.pdf]

NC ← *D’* – *OC*;   // *NC* denotes the set of new cases in *D’*

D’ ← *D’* – *NC*;

Grouping(*NC, G, OC, k,* θ^*); // Stage 2

R_i ← *Generalization*(*D’* + *NC, G, k,* θ^*); // Stage 3

return R_i;
