# Supplementary material for: Privacy-Preserving Anonymity for Periodical Releases of Spontaneous Adverse Drug Event Reporting Data: Algorithm Development and Validation
Source: JMIR Med Inform. 2021 Oct 28;9(10):e28752. doi: 10.2196/28752 (PMC8587328; doi:10.2196/28752)
Supplement: Multimedia Appendix 10 [file medinform_v9i10e28752_app10.pdf]

The PRR measurement requires the following 2×2 contingency table,

| Predc | *symptom* | other *symptom*s |
|---|---|---|
| *drug* | *a* | *b* |
| other *drug*s | *c* | *d* |

where under the extra conditions, *a* is the number of reports which contain the suspected drug and reaction, *b* is the number of reports containing the suspected drug but not the suspected reaction, *c* is the number of reports containing the suspected reaction but not the suspected drug, and *d* is the number of reports containing neither the drug nor the suspected reaction. The definition of PRR is as follows:

$$PRR = \frac{a/(a+b)}{c/(c+d)}$$
